# Supplementary material for: Isolation and Characterization of Cell Envelope Fragments Comprising Archaeal S-Layer Proteins
Source: Nanomaterials (Basel). 2022 Jul 21;12(14):2502. doi: 10.3390/nano12142502 (PMC9320373; doi:10.3390/nano12142502)
Supplement: Supplementary file 1 [file nanomaterials-12-02502-s001.zip › nanomaterials-1788760-supplementary.pdf]

nanomaterials

MDPI

Supplementary Materials

Isolation and Characterization of Cell Envelope Fragments Comprising Archaeal S-Layer Proteins

Kevin Pfeifer [1], Eva-Kathrin Ehmoser [1], Simon K.-M. R. Rittmann [2,3], Christa Schleper [4], Dietmar Pum [5], Uwe B. Sleytr [1] and Bernhard Schuster [1,*]

[1] Institute of Synthetic Bioarchitectures, University of Natural Resources and Life Sciences, 1190 Vienna, Austria; kevinpfeifer@boku.ac.at (K.P.); eva.ehmoser@boku.ac.at (E.-K.E.); uwe.sleytr@boku.ac.at (U.B.S.); bernhard.schuster@boku.ac.at (B.S.)
[2] Archaea Physiology & Biotechnology Group, Department of Functional and Evolutionary Ecology, University of Vienna, 1030 Vienna, Austria; simon.rittmann@univie.ac.at
[3] Arkeon GmbH, 3430 Tulln an der Donau, Austria
[4] Archaea Biology and Ecogenomics Division, Department of Functional and Evolutionary Ecology, University of Vienna, 1030 Vienna, Austria; christa.schleper@univie.ac.at
[5] Institute of Biophysics, University of Natural Resources and Life Sciences, 1190 Vienna, Austria; dietmar.pum@boku.ac.at
* Correspondence: bernhard.schuster@boku.ac.at; Tel.: +43-1-47654-80436

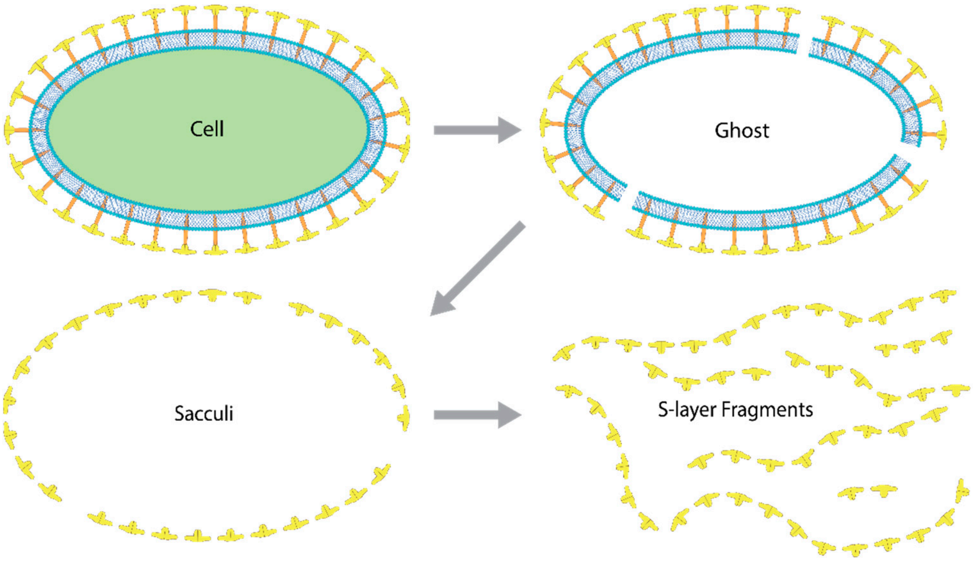

Figure S1. Sequential steps and terminologies throughout the S-layer protein extraction process. Simplified model of a *Saccharolobus solfataricus* P2 (SSO) cell showing the sequential steps and terminologies throughout the S-layer protein (SLP) extraction process. SSO cells are lysed, and their cytosolic content (green) removed to produce ghosts consisting of a cytoplasmic membrane (CM) (blue), membrane associated proteins (MP) and the S-layer components SlaA (yellow) and SlaB (orange). To produce sacculi, the ghosts are washed with detergent to remove CM, MP, and SlaB from the crystalline SlaA lattice. The sacculi are finally sonicated to produce SSO S-layer fragments comprising of SlaA.

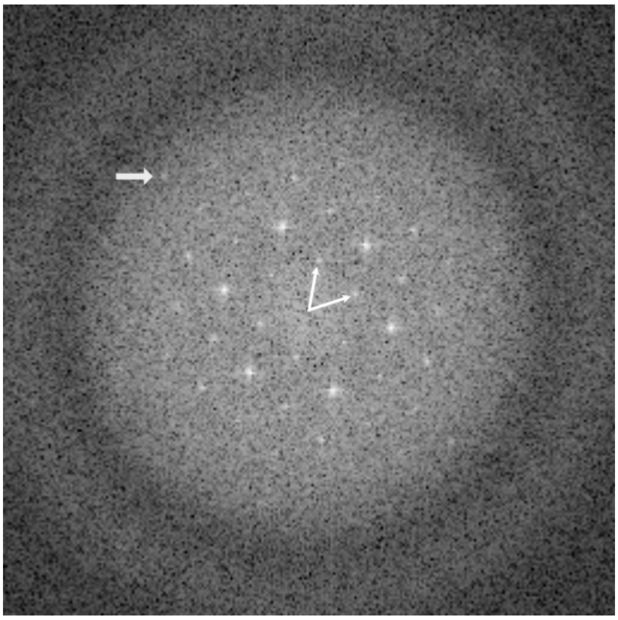

Figure S2. Fourier spectrum of a TEM image of an *Saccharolobus solfataricus* P2 fragment. The central part of the Fourier spectrum of a TEM image of an *Saccharolobus solfataricus* P2 (SSO) fragment is shown. The base vectors of the reciprocal lattice are indicated by arrows. The highest order spot (-4/4), corresponding to a resolution of 4.5 nm, is marked as well. The first zero crossing of the contrast transfer function (CTF) and the first Thon fringe are clearly visible. The image processing routines were developed in-house as plugins for the open-source software ImageJ. This figure was obtained by standard ("optical") filtering. A digital version of the well-known optical mask used in diffractometers in the 1960's and 1970's was generated based on the reciprocal lattice which had been identified after indexing the peaks in the Fourier spectrum. Peaks were considered only if their intensity was higher than a threshold of 1.5 with respect to the average intensity of peaks in a surrounding area of 9x9 pixels. The diameter of the "punched" holes in the digital filter mask was set to 20% of the length of the basis vector. A cosine^2-function was used to smooth the edge of the filter holes. The reconstructed image was finally obtained by applying the filter mask to the digital Fourier transform and performing the inverse Fourier transform.

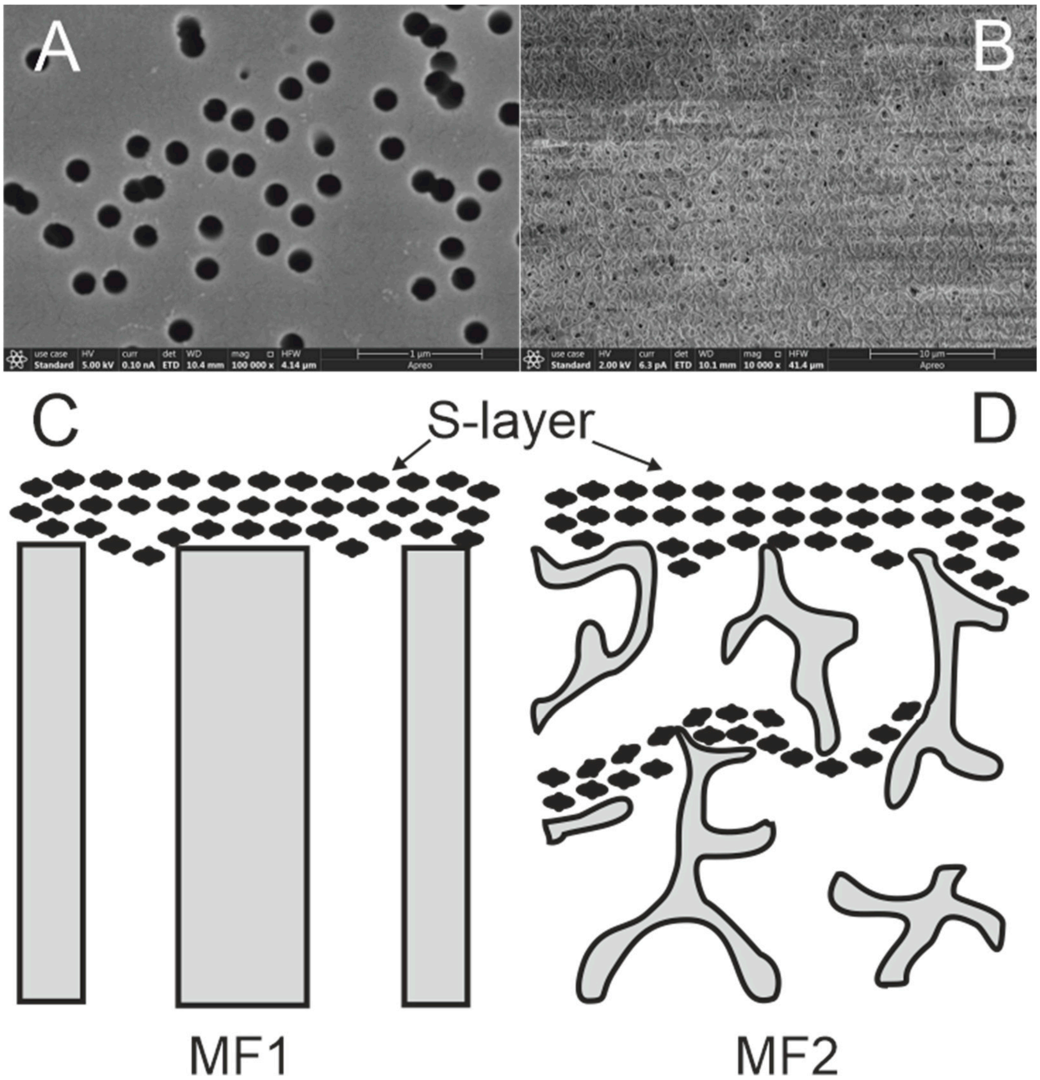

Figure S3. Types of microfiltration membranes and attachment of S-layer fragments. **A)** SEM image (top view) of a radiation-track membranes (MF1) with 100.000x magnification. **B)** SEM image (top view) of an open-celled foam-like microfiltration membranes (MF2) with 10.000x magnification. **C)** Schematic drawing of S-layer fragments, which are attached to the surface of MF1. Indicated are also how the pores in MF1 look like from side view. **D)** Schematic drawing of S-layer fragments, which are deposited on the surface and in the substructure of MF2. Indicated are also how the pores in MF2 look like from side view. Modified after Ref. [38]. Copyright © 2022 with permission from Elsevier Science Publishers B.V., Amsterdam.

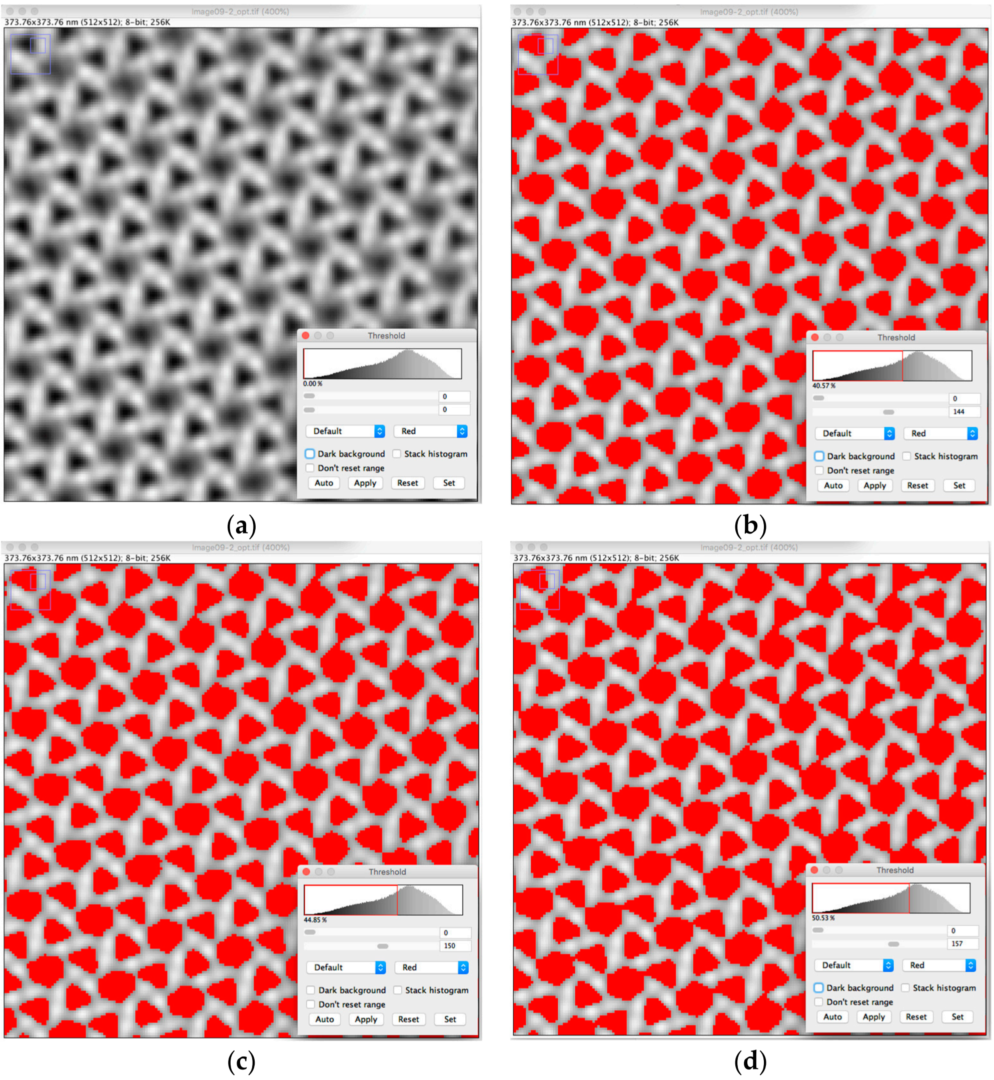

Figure S4. SDS-PAGE of a protein mixture filtered through *Saccharolobus solfataricus* P2 fragments. SDS-PAGE (4-20% Bis-Tris) of a mixture of proteins filtered through *Saccharolobus solfataricus* P2 fragments, which have been deposited on microfilter 1 (SSOMF1). F1-F3 = 3 Biological filtration replicates; R1-R3 = Corresponding retentate; C = Original protein mixture; and a reference gel showing each protein stock solution run in its own lane. Proteins used for filtration: myoglobin (MyG; 17 kDa); carbonic anhydrase (CA; 31 kDa); horseradish peroxidase (PO; 44 kDa); bovine serum albumin (BSA; 66 kDa); amyloglucosidase (AmG; 97 kDa).

Table S1. Cultivation Cost Calculation.

**500mL Medium**						
	mL	€				
50% H_2SO_4 (mL)	0,135	€ 0,02				
Sucrose 10%	5	€ 0,02				
Yeast 10%	5	€ 0,11				
100 Brock	5	€ 0,15				
200 Brock	2,5	€ 0,09				
1000 Brock	0,5	€ 0,00				
		**€ 0,39**				
**100 Brock**	g/L	€/L	€/mL			
$(NH_4)_2SO_4$	130	€ 23,06	€ 0,02			
$MgSO_4$ x7 H_2O	25	€ 5,45	€ 0,01			
$FeCl_3$ x 6 H_2O	2	€ 0,36	€ 0,00			
50% H_2SO_4 (ml)	3	€ 0,94	€ 0,00			
		**€ 29,81**	**€ 0,03**			
**200 Brock**	g/L	10mg/mL (mL)	mg	g	€/L	€/mL
KH_2PO_4	56				€ 34,94	€ 0,03
$MnCl_2$		3,6	36	0,036	€ 0,10	€ 0,00
$ZnSO_4$		4,4	44	0,044	€ 0,03	€ 0,00
$CuCl_2$		1	10	0,01	€ 0,08	€ 0,00
$VOSO_4$		0,6	6	0,006	€ 0,08	€ 0,00
$CoSO_4$		0,2	2	0,002	€ 0,00	€ 0,00
$Na_2B_4O_7$		0,09	0,9	0,0009	€ 0,00	€ 0,00
Na_2MoO_4		0,6	6	0,006	€ 0,00	€ 0,00
					**€ 35,24**	**€ 0,04**
**1000 Brock**	g/L	€/L	€/mL			
$CaCl_2$ x 2 H_2O	14	**€ 2,21**	**€ 0,00**			
**Yeast**	g/L					
Yeast 10%	100	**€ 21,60**	**€ 0,02**			
**Sucrose**	g/L					
10%	100	**€ 3,40**	**€ 0,00**			
**Chemicals**	Order Quantity (g)	Price (€)	Price/g (€)			
(NH4)2SO4	500	€ 88,70	€ 0,18			
MgSO4 x7H2O	500	€ 109,00	€ 0,22			
FeCl3 x 6H2O	250	€ 44,90	€ 0,18			
KH_2PO_4	250	€ 156,00	€ 0,62			
$MnCl_2$	50	€ 137,00	€ 2,74			
$ZnSO_4$	100	€ 64,90	€ 0,65			
$CuCl_2$	25	€ 204,00	€ 8,16			
$VOSO_4$	10	€ 135,00	€ 13,50			
$CoSO_4$	100	€ 84,90	€ 0,85			
Na_2B4O_7	500	€ 76,50	€ 0,15			
Na_2MoO_4	100	€ 63,30	€ 0,63			
$CaCl_2$ x2 H_2O	500	€ 79,10	€ 0,16			
Yeast Extract	1000	€ 216,00	€ 0,22			
Sucrose	1000	€ 34,00	€ 0,03			
H_2SO_4	1000	€ 342,00	€ 0,34			

Table S2. Extraction Cost Calculation.

Buffer A SEG		g in 500mL	€ for 500mL	€ for 1mL
NaCl	10mM	0,3	€ 0,03	€ 0,00
N-Lauroylsarcosin sodium salt	0,50%	2,5	€ 3,04	€ 0,01

			€ 3,07	€ 0,01
Buffer A DEG		g in 500mL	€ for 500mL	€ for 1mL
NaCl	10mM	0,3	€ 0,03	€ 0,00
N-Lauroylsarcosin sodium salt	0,50%	2,5	€ 3,04	€ 0,01
PMSF	1mM	0,09	€ 1,51	€ 0,00
			€ 4,58	€ 0,01
Buffer B		g in 500mL	€ for 500mL	€ for 1mL
NaCl	10mM	0,3	€ 0,03	€ 0,00
Sodiumdodecylsulfate	0,50%	2,5	€ 4,84	€ 0,01
Magnesiumsulfate	0.5mM	0,03	€ 0,01	€ 0,00
			€ 4,88	€ 0,01
Extraction of 500ml Culture (SEG)				
	mL or mg	Repeat Steps	Total mL	Total €
Buffer A	100	1	100	€ 0,61
Buffer B	25	4	100	€ 0,98
				€ 1,59
Extraction of 500ml Culture (DEG)				
	mL or mg	Repeat Steps	Total mL	Total €
Buffer A	400	1	400	€ 3,67
Buffer B	15	4	60	€ 0,59
DNAse 1 10µg/ml (mg)	4	1	4	€ 5,92
				€ 10,17
Extraction of 500ml Culture (DEG)				
	mL	Repeat Steps	Total mL	Total €
Buffer A	400	1	400	€ 3,67
Buffer B	15	4	60	€ 0,59
Turbo DNAse units	100	1	100	€ 16,30
				€ 20,55
Cost per mg S-layer protein				
	50mL	500mL (calculated)	Extraction Cost	Extraction Cost/mg
Sonicated	0,97	0,97	€ 0,16	€ 0,16
Sonicated	1,03	1,03	€ 0,16	€ 0,15
Sonicated	0,88	0,88	€ 0,16	€ 0,18
DNAse Digested	1,32	1,32	€ 1,02	€ 0,77
DNAse Digested	1,17	1,17	€ 1,02	€ 0,87
DNAse Digested	1,14	1,14	€ 1,02	€ 0,89
Cost per mg S-layer protein				
	Cost/mg S-layer average	Relative cost average	Cost/mg S-layer StDv	Relative cost StDv
Sonicated	€ 0,17	18%	€ 0,01	€ 0,01
DNAse Digested	€ 0,84	100%	€ 0,05	€ -

Reference

38. Sára, M.; Sleytr, U.B. Production and characteristics of ultrafiltration membranes with uniform pores from two-dimensional arrays of proteins. *J. Membr. Sci.* **1987**, *33*, 27–49.
